# Appropriateness of strategy comparisons in cost-effectiveness analyses of infant pneumococcal vaccination: a systematic review

**DOI:** 10.1017/S0266462323000351

**Published:** 2023-07-12

**Authors:** Mariska M. J. Scheffer, Luc E. Coffeng, James F. O’Mahony

**Affiliations:** 1 Department of Public Health, Erasmus MC, University Medical Center Rotterdam, Rotterdam, The Netherlands; 2 Department of Care and Participation of People with Chronic Conditions, Nivel (Netherlands Institute for Health Services Research), Utrecht, The Netherlands; 3 Centre for Health Policy and Management, Trinity College Dublin, Dublin, Ireland

**Keywords:** comparator selection, economic evaluation, health outcomes, pneumococcal disease, vaccines

## Abstract

**Objectives:**

Cost-effectiveness analysis (CEA) is the standard framework for informing the efficient allocation of scarce healthcare resources. The importance of considering all relevant intervention strategies and appropriate incremental comparisons have both long been recognized in CEA. Failure to apply methods correctly can lead to suboptimal policies. Our objective is to assess if CEAs of infant pneumococcal vaccination apply appropriate methods with respect to the completeness of strategies assessed and incremental comparisons between them.

**Methods:**

We conducted a systematic search of the PubMed, Scopus, Embase, and Web of Science databases and performed a comparative analysis of the retrieved pneumococcal vaccination CEAs. We checked the appropriateness of the incremental analyses by attempting to replicate the published incremental cost-effectiveness (CE) ratios from the reported costs and health effects.

**Results:**

Our search returned twenty-nine eligible articles. Most studies failed to recognize one or more intervention strategies (*n* = 21). Incremental comparisons were questionable in four CEAs and insufficient reporting of cost and health effect estimates was identified in three studies. Overall, we only found four studies that made appropriate comparisons between all strategies. Lastly, study findings appear to be strongly associated with manufacturer sponsorship.

**Conclusions:**

We found considerable scope for improvement regarding strategy comparison in the infant pneumococcal vaccination literature. To prevent overestimation of the CE of new vaccines, we urge greater adherence to existing guidelines recommending that all available strategies are evaluated to capture relevant comparators for CE evaluation. Closer adherence to existing guidelines will generate better evidence, leading to more effective vaccination policies.

## Introduction

Pneumococcal disease is major communicable cause of morbidity and mortality, leading to approximately 1.2 million annual deaths worldwide in the absence of vaccination (1;2). Infection leads to various conditions: noninvasive pneumococcal diseases (sinusitis, acute otitis media, and community-acquired pneumonia); or, invasive pneumococcal diseases (meningitis and bacteremia) (1). While everyone is susceptible to infection, very young children, older adults, and those with certain comorbidities face higher risks (1).

Several vaccines for pneumococcal disease have been introduced, including those suitable for infants and children. These can be divided into conjugate and polysaccharide vaccines (3). The first commonly adopted vaccine was the 23-valent pneumococcal polysaccharide vaccine (PPSV23, Pneumovax 23®). It is not immunogenic in young children (4). The more recently introduced 7-valent (serotypes 4, 6B, 9 V, 14, 18C, 19F and 23F) pneumococcal conjugate vaccine (PCV7, Prevenar®) is effective in infants and children (3). Most recently, the 10-valent and 13-valent pneumococcal conjugate vaccines (PCV10, Synflorix**™** and PCV13, Prevenar 13®, respectively) were developed. PCV10 includes the 7-valent vaccine serotypes plus the 1, 5, and 7F serotypes, whereas PCV13 additionally includes serotypes 3, 6A, and 19A (3;5).

The availability of multiple vaccinations has implications when conducting a health economic evaluation to find the most cost-effective prevention strategy. In order to select the most appropriate health policy, a central premise of cost-effectiveness analysis (CEA) methods is that analyses include all feasible alternatives (6). Another important, long-recognized methodological principle within CEA is that strategies are compared to each other incrementally, meaning that strategies are compared to the next best alternative intervention, as opposed to no intervention at all (7). Such comparisons are required for the correct estimation of incremental cost-effectiveness ratios (ICERs). Accordingly, the inclusion of all relevant strategies is important both not only for the assessment of all relevant candidate strategies, but also for the inclusion of sufficient comparator strategies against which to compare candidate strategies. The omission of sufficient comparator strategies can result in biased ICERs, making interventions appear more cost-effective than they are.

Prior research has raised concerns about inappropriate application of CEA methods within the pneumococcal vaccination literature. A systematic review of CEAs of pneumococcal interventions in low- and middle-income countries (LMICs) noted that many did not consider all potential strategies (no vaccination, PCV7, PCV10, or PCV13) and sometimes failed to make appropriate incremental comparisons (8). It was shown that most studies reported the cost-effectiveness (CE) of vaccines versus no-vaccination, rather than using a correct incremental approach. These observations are consistent with findings of a recent research on pneumococcal vaccination for children in Asian countries, which also found omission of relevant strategies (9).

Despite a relatively large body of CEA evidence regarding infant pneumococcal vaccination, there is no clear consensus on which vaccination is optimally cost-effective. These conflicting conclusions regarding CE leaves decision makers facing uncertainty what strategy to adopt. We are assessing to what extent the absence of consensus among CEAs of infant pneumococcal vaccination is potentially due to inappropriate strategy comparisons. In contrast to previous research which focused on LMICs (8;9), our analysis reviews the evidence of economic evaluations of infant pneumococcal vaccination in high-income countries (HICs). To the best of our knowledge, this is the first study to specifically assess questions of strategy comparison in this context. We conducted a systematic review followed by a critical assessment of the recovered studies to gain insight into the reliability of policy recommendations.

## Methods

### Search strategy

We searched the PubMed, Scopus, Embase, and Web of Science electronic databases for health economic evaluations of infant pneumococcal vaccination. Our search strings are presented in Appendix I of the Supplementary Material. The database searches were restricted to scientific journal articles published since 2010. This was the year after the introduction of PCV10, from when the choice between multiple vaccination strategies became relevant. The search was limited to articles published in English and was conducted on 15 June 2022.

### Selection process

As a first step, we screened and selected candidate articles based on title and abstract. Our inclusion and exclusion criteria can be understood within the population, intervention, comparator, outcomes, and study (PICOS) framework as follows (10). The population of interest are the general infant population from HICs, as defined by the World Bank classification (11). We excluded non-infant populations and specific groups of elevated risk. The interventions of interest are vaccination against pneumococcal disease of all vaccine types. We excluded articles if they were not related to interventions against pneumococcal disease. The question of what strategies were used as comparators is embedded in our research question and so did not constitute part of our inclusion and exclusion criteria. Regarding outcomes and study type, the inclusion criteria were CEAs reporting outcomes in terms of cost per quality-adjusted life-years (QALYs) or life-years gained (LYG). As a corollary, we excluded study designs that were not health CEAs; which may include reviews, methodological studies, and cost of illness studies. As such, we excluded studies reporting health economic outcomes other QALY or LYG, such as the cost per case prevented.

### Data collection and analysis

As our review was focused on the specific question of whether CEAs had included sufficient intervention strategies and compared them appropriately, we did not apply a general quality appraisal tool such as the CHEERS or Drummond and Jefferson checklists (12;13). Instead, we used our own critical appraisal framework to assess the reliability of each study’s policy recommendations with respect to three criteria: (i) did the analysis report all costs and effects transparently; (ii) did it include all relevant comparator strategies; and (iii) did it perform appropriate incremental comparisons between the costs and effects of the alternative strategies? We explain these in greater detail in the paragraphs below. We considered policy recommendations to be reliable once all three criteria were met. In addition, we recorded the funding reported by each study. Finally, to assess the heterogeneity of identified CEAs, we compiled the costs and effects of all studies in summary CE planes.

#### Transparency of reporting

The premise of this assessment is that estimated costs and health effects should be reported with sufficient clarity to permit replication of the reported CE estimates. We assessed if studies reported estimates numerically for both costs and effects over all simulated strategies. When the costs and effects were reported, we determined if it was possible to reproduce the reported ICERs.

#### Inclusion of relevant comparators

The relevant strategies in the context of infant pneumococcal vaccination currently are the no-vaccination strategy, and immunization with PCV7, PCV10, or PCV13. As the vaccines were introduced at different points in time, what vaccines constitute relevant comparators at any prior point in time depends on the year of licensing (Appendix II of the Supplementary Material). We considered the omission of a vaccine type in a study published 2 years or more after the type was licensed to be the exclusion of a relevant comparator. The omission of available intervention strategies might also be justified in certain circumstances. For example, if a strategy has been clearly demonstrated as inefficient in prior studies and cited as such, then it may be justified for omission in future analyses. Accordingly, we examined whether a rationale for the exclusion of an omitted strategy was given within the reviewed studies.

#### Appropriateness of incremental comparisons

We assessed the appropriateness of incremental comparisons within the published estimates from each CEA. A correct ICER estimate is understood to be the incremental comparison of the differences in costs and effects between strategies that lie on the efficient frontier, with a given intervention being compared to the next most effective efficient strategy (6). We evaluated if the reported ICERs were based on the accepted understanding of ICERs or were based on other comparisons, such as those that simply compared to no vaccination or compared to another vaccine strategy that was not the adjacent strategy on the efficient frontier.

#### Heterogeneity of estimates

We conducted an assessment to determine how heterogeneous the cost and health effects estimates were over the simulated strategies between the reviewed studies. We plotted the CEA results in a series of summary CE planes. These summary CE planes do not report numerical costs and health effects, but give a qualitative summary of whether a given strategy was dominant, dominated, cost-effective or not when compared to a specific comparator. We used a series of these CE planes to summarize the results of different strategy comparisons across multiple studies. Dominant strategies are those with greater effects and lower costs than the reference strategy. These are presented within the south east quadrant of the summary CE planes. Conversely, strategies that are dominated, both more expensive and less effective, are depicted in the northwest quadrant. The north east and south west quadrants are divided between those interventions that are cost-effective or not. We determined the CE with respect to the CE threshold cited within each individual CEA.

#### Sponsorship association

We also examined the association between reported research sponsorship and the comparator choice and study conclusions. We categorized the data to facilitate quantitative analysis: this included binary categories regarding whether PCV7 was included; whether the no-vaccination strategy was included; whether PCV13 or PCV10 (latest vaccines) was recommended when compared; whether the policy recommendation was considered reliable according to the three criteria described above; and whether the research was reported as funded by a pharmaceutical company. We analyzed the data using STATA Software for Statistics and Data Science (Version 16.1). We applied Fisher’s exact test for the evaluation of categorical data, which is accurate when using a small sample for analysis (14). A *p*-value lower than .05 was considered statistically significant.

## Results

### Description of identified CEAs

**Figure 1.**
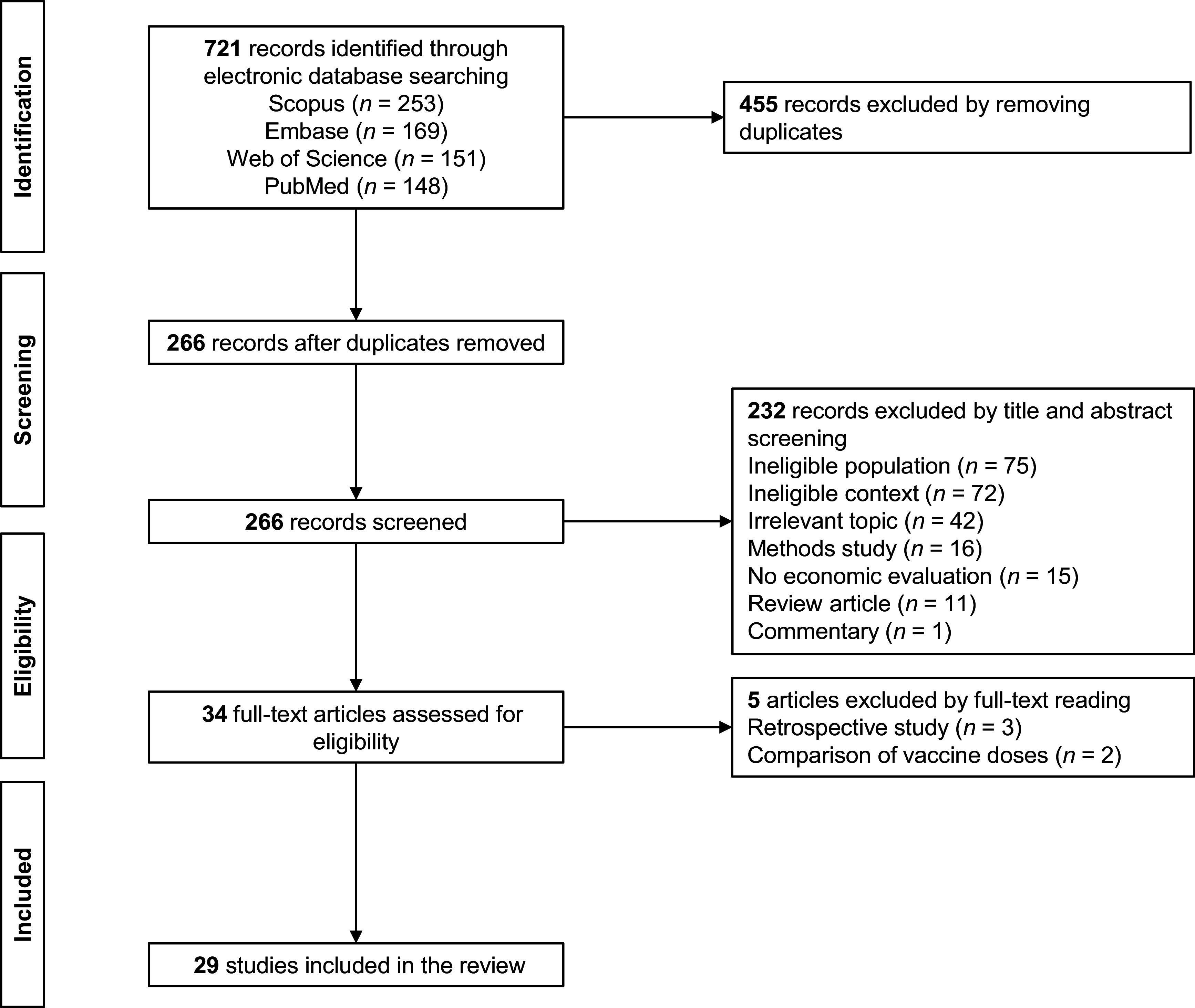
Flow diagram of review process.

As presented in Figure 1, the databases Scopus, Embase, Web of Science, and PubMed returned 253, 169, 151, and 148 articles, respectively. Following pooling and removal of duplicates, we retrieved unique 266 articles for title and abstract screening. Our first screen identified thirty-four studies for full-text screening. Subsequently, we removed three studies from analysis as they differed in study type (retrospective cohorts) (15–17). We excluded another two studies as they focused on dosing schedules for one pneumococcal vaccine alone (18;19). We extracted data from the remaining studies, including the publication year, national setting, vaccine intervention, chosen comparators, sponsorship, cost and effect estimates, reported ICERs, and related policy recommendations. In total, we retained twenty-nine CEAs of infant pneumococcal vaccination for further analysis (20–48).

An overview of the twenty-nine eligible studies is presented in Table 1. The date of publication ranged from 2010 to 2020. The CEAs were conducted in twenty different countries. The number of intervention options varied between studies, ranging from two to four strategies (including no vaccination). The no-vaccination strategy, PCV7, PCV10, and PCV13 vaccines were included in 13, 11, 21, and 26 studies, respectively. Regarding sponsorship, twenty-two studies reported funding from pharmaceutical companies. Of these sponsored studies, eleven reported funding from Pfizer (formerly Wyeth), which manufactures the PCV7 and PCV13 vaccines. Eleven studies reported sponsorship by GlaxoSmithKline (GSK), which manufactures PCV10. Most CEAs included QALYs as a health outcome measure (*n* = 25, 86 percent).

**Table 1.** Overview identified economic evaluations of childhood pneumococcal vaccination strategies (*n* = 29)

First author		Year	Setting	Measure	Financial support	NV	PCV7	PCV10	PCV13	Most effective strategy	Most cost-effective strategy
1	Rozenbaum	2010	Netherlands	€/QALY	Wyeth (Pfizer)	×	×	×	×	PCV13	PCV13
2	Rubin	2010	United States	$/QALY $/LYG	Pfizer	–	×	–	×	PCV13	PCV13
3	Sohn	2010	South Korea	KW/LYG	Independent	×	×	–	–	PCV7	NV
4	Díez-Domingo	2011	Spain	€/QALY€/LYG	Pfizer	×	–	–	×	PCV13	PCV13
5	Knerer	2011	Canada, United Kingdom	€/QALY €/LYG	GSK	–	–	×	×	PCV10	PCV10
6	Newall	2011	Australia	$/QALY	GSK	×	×	×	×	Unclear	Unclear
7	Robberstad	2011	Norway	NOK/QALY €/QALY	GSK	×	×	×	×	PCV10	PCV10
8	Tyo	2011	Singapore	$/QALY	Independent	×	×	×	×	PCV13	PCV13
9	Blank	2012	Switzerland	€/QALY €/LYG	Pfizer	–	×	–	×	PCV13	PCV13
10	By	2012	Sweden	SEK/QALY	GSK	×	–	×	×	PCV10	PCV10
11	Earnshaw	2012	Canada	$/QALY	Pfizer	–	–	×	×	PCV13	PCV13
12	Hoshi	2012	Japan	JPY/QALY	Independent	×	×	–	–	PCV7	PCV7
13	Hoshi	2013	Japan	JPY/QALY	Independent	×	×	–	×	PCV13	PCV13
14	Klok	2013	Denmark, Sweden	DKK/QALYSEK/QALY	Pfizer	–	–	×	×	PCV13	PCV13
15	Lee	2013	Hong Kong	HKD/QALY HKD/LYG	GSK	–	–	×	×	PCV10	PCV10
16	Wu	2013	Taiwan	NT$/LYG	Independent	×	×	–	–	PCV7	PCV7
17	Vemer	2014	Netherlands	€/QALY	GSK	–	–	×	×	PCV13	PCV13
18	Shiragami	2015	Japan	JPY/QALY	GSK	–	–	×	×	PCV10	PCV10
19	Vučina	2015	Croatia	$/DALY	Independent	×	–	×	×	PCV13	NV
20	Delgleize	2016	United Kingdom	£/QALY	GSK	–	–	×	×	PCV10	PCV10
21	Castiglia	2017	Italy	€/QALY	GSK	×	×	×	×	PCV10	PCV10
22	Gouveia	2017	Portugal	€/YLL	Pfizer	×	–	–	×	PCV13	PCV13
23	Kuhlmann	2017	Germany	€/LYG €/QALY	Independent	–	–	×	×	PCV13	PCV13
24	Wilson	2018	Canada	$/LYG $/QALY	Pfizer	–	–	×	×	PCV13	PCV13
25	Zhang	2018	South Korea	$/QALY	GSK	–	–	×	×	PCV10	PCV10
26	Ansaldi	2020	Italy	€/QALY	Pfizer	–	–	×	×	PCV13	PCV13
27	Kim	2020	South Korea	$/QALY	Pfizer	–	–	×	×	PCV13	PCV13
28	Lu	2020	Taiwan	NT$/QALY	GSK	–	–	×	×	PCV10	PCV10
29	Pugh	2020	Finland, Netherlands	€/LYG €/QALY	Pfizer	–	–	×	×	PCV13	PCV13

Abbreviations: DKK, Danish krone; GSK, GlaxoSmithKline; HKD, Hong Kong dollar; JPY, Japanese yen; KW, Korean Republic won; NOK, Norwegian krone; NT$, New Taiwan dollar; NV, no vaccination; SEK, Swedish krona.

Table 1 shows there is a marked lack of consensus regarding which vaccine is optimally cost-effective. To illustrate, of four CEAs that included all strategies, two reported that PCV10 was most cost-effective (29;42), while another two concluded that PCV13 had more cost-effective outcomes (30;35). The majority of identified optimal strategies were dominant in that they are estimated to be more effective and less costly than alternative strategies (*n* = 18, 62 percent). Of the twenty-two studies that reported industry sponsorship, twenty-one concluded that the sponsor’s product was the most cost-effective.

### Transparency of reporting

Three studies reported incomplete estimates (27;30;35). Of these, one failed to report the total net costs of immunization programs (or constituent cost components from which they could be derived) (30). The second study did not report the total net costs, and although it report the direct and indirect savings of interventions, it excluded the vaccination costs, again precluding the derivation of total net costs (35). The third study did not clearly report all costs and health outcomes for each vaccination strategy (27). Consequently, we could not reproduce the complete CE plane and ICERs for these studies.

### Inclusion of relevant comparators

Of the twenty-nine identified CEAs, only five considered all possible strategies (no-vaccination, PCV7, PCV10, and PCV13; Table 1) (27;29;30;35;42). From the studies that omitted one or more strategies, twenty excluded either the no-vaccination or PCV7 strategy (20–26;28;31–33;36–38;40;41;43–46). We identified four PCV10 omissions (21;39;46;47) and one PCV13 omission that were not justified by a supporting rationale for exclusion (39). Cases of justified exclusions included instances where vaccines were not licensed at the time the study was conducted; the exclusion of PCV10 and PCV13 was considered justified in two studies on this basis (34;48). More broadly, we interpreted the omission of the no-vaccination strategy as justified, as most of the identified studies showed that pneumococcal vaccines are cost-effective in HICs. Nevertheless, we interpreted the omission of PCV7 as unjustified, as there is limited evidence that this strategy is dominated by other PCVs. Moreover, there were five CEAs of either PCV10 or PCV13 that omitted the other type (21;31;44;46;47). As there was no consensus on the CE of PCV10 and PCV13, the exclusion of either one was considered not justified.

### Appropriateness of incremental comparisons

There were eighteen studies (62 percent) that found one of pneumococcal vaccine types to dominate the other strategies and so had no ICERs to report (21–24;26;28;29;31–33;38;40–43;45–47). Within the other eleven studies, the ICER estimates were questionable in four evaluations (36 percent) (25;27;30;35). Two compared candidate strategies to the no-vaccination strategy alone rather than making incremental comparisons to other vaccine strategies (30;35). Similarly, another study compared all possible vaccination strategies to the no-vaccination and PCV7 strategies, the latter of which was current practice (27). Finally, one study compared both PCV10 and PCV13 to PCV7 rather than conducting a standard incremental analysis that would have shown PCV10 to be subject to extended dominance (25).

### Overall appropriateness of comparisons

Table 2 provides a summary of the three assessment criteria of clear reporting of costs and effects, relevant comparator inclusion and appropriate incremental comparisons as applied to the reviewed studies. It also provides our summary judgment regarding the appropriateness of the comparisons based on these three criteria. Of the twenty-nine CEAs, four (14 percent) presented analyses that were judged to be fully appropriate (29;34;42;48). The remaining evaluations were found to contain shortcomings, including twenty-one (72 percent) that omitted one or more relevant comparators without justification, leading our conclusion that their comparisons were not fully appropriate (20–24;26;28;31–33;36–41;43–47). In addition, four failed to report cost and effects adequately and/or make appropriate incremental analyses, leading to our conclusion that their comparisons were not appropriate (25;27;30;35).

**Table 2.** Summary critical appraisal of cost-effectiveness analyses (*n* = 29)

First author (year)	Costs and effects reported	Comparators excluded	Comparator exclusion justified	Appropriate incremental comparison	Adequacy of comparisons
Rozenbaum (2010)	Incomplete	Probably not	NA	No	Inadequate
Rubin (2010)	Complete	Probably	Unjustified	NA	Partial
Sohn (2010)	Complete	Probably	Justified	Yes	Adequate
Díez-Domingo (2011)	Complete	Probably	Unjustified	Yes	Partial
Knerer (2011)	Complete	Probably	Unjustified	NA	Partial
Newall (2011)	Incomplete	Probably not	NA	No	Inadequate
Robberstad (2011)	Complete	Probably not	NA	NA	Adequate
Tyo (2011)	Incomplete	Probably not	NA	No	Inadequate
Blank (2012)	Complete	Probably	Unjustified	NA	Partial
By (2012)	Complete	Probably	Unjustified	NA	Partial
Earnshaw (2012)	Complete	Probably	Unjustified	NA	Partial
Hoshi (2012)	Complete	Probably	Justified	Yes	Adequate
Hoshi (2013)	Complete	Probably	Unjustified	NA	Partial
Klok (2013)	Complete	Probably	Unjustified	NA	Partial
Lee (2013)	Complete	Probably	Unjustified	NA	Partial
Wu (2013)	Complete	Probably	Unjustified	Yes	Partial
Vemer (2014)	Complete	Probably	Unjustified	Yes	Partial
Shiragami (2015)	Complete	Probably	Unjustified	NA	Partial
Vučina (2015)	Complete	Probably	Unjustified	Yes	Partial
Delgleize (2016)	Complete	Probably	Unjustified	NA	Partial
Castiglia (2017)	Complete	Probably not	NA	NA	Adequate
Gouveia (2017)	Complete	Probably	Unjustified	NA	Partial
Kuhlmann (2017)	Complete	Unclear	Unclear	No	Inadequate
Wilson (2018)	Complete	Probably	Unjustified	NA	Partial
Zhang (2018)	Complete	Probably	Unjustified	NA	Partial
Ansaldi (2020)	Complete	Probably	Unjustified	Yes	Partial
Kim (2020)	Complete	Probably	Unjustified	NA	Partial
Lu (2020)	Complete	Probably	Unjustified	NA	Partial
Pugh (2020)	Complete	Probably	Unjustified	NA	Partial
**Total**	**Three incomplete reporting**	**Twenty-three excluded comparators**	**Twenty-one unjustified choice of comparators**	**Four inappropriate incremental comparisons**	**Four adequate comparisons**

Abbreviation: NA, not applicable.

### Heterogeneity of results

**Figure 2.**
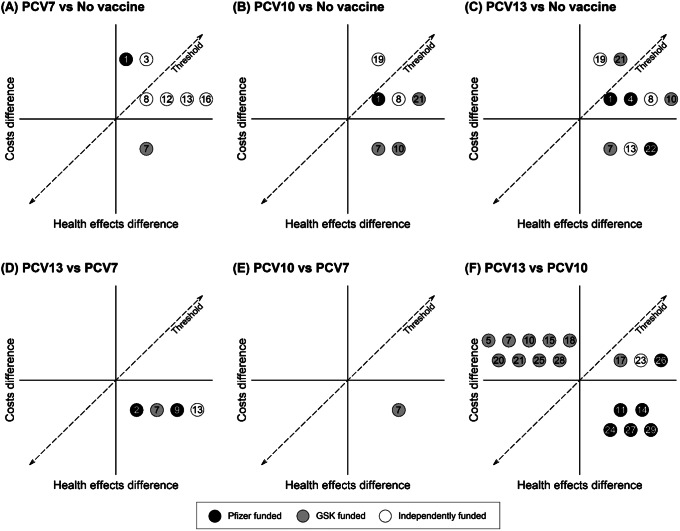
Overview cost-effectiveness comparison of pneumococcal conjugate vaccines. The qualitative summary planes are shown for each pair of vaccine strategies: PCV7 and no-vaccination (2A), PCV10 and no-vaccination (2B), PCV13 and no-vaccination (3C), PCV13 and PCV7 (3D), PCV10 and PCV7 (3E), PCV13 and PCV10 (3F). GSK, GlaxoSmithKline; PCV7, 7-valent pneumococcal conjugate vaccine; PCV10, 10-valent pneumococcal conjugate vaccine; PCV13, 13-valent pneumococcal conjugate vaccine.

Figure 2 presents the qualitative summary CE planes for each pair of vaccine strategies according to each study as numbered in Table 1. Each number qualitatively records the relative CE of each of the strategy comparisons as reported by the respective studies. For example, Figure 2A shows all the studies that compared PCV7 and no vaccination found PCV7 to be effective. Studies 1 and 3 found PCV7 to be net costly and cost-ineffective, 8, 12, 13 and 16 found it to be net costly and cost-effective, while 7 found it to be cost-saving and thus dominant. Figure 2F reveals largely contradictory findings of dominance among those studies comparing PCV10 to PCV13. Almost all studies funded by Pfizer comparing PCV13 to PCV10, concluded that PCV13 is dominant. Conversely, most studies funded by GSK found that PCV13 is dominated by PCV10. Not all studies favor their own vaccine, one study found the rival’s product is more cost-effective (36). Nonetheless, the summary CE planes show considerable heterogeneity regarding which strategy is estimated the most cost-effective.

### Sponsorship association

Our results indicate that a greater proportion of industry-sponsored studies excluded PCV7 (*n* = 16/22, 73 percent) relative to independent studies (*n* = 2/7, 29 percent), however, this is not a statistically significant difference (*p* = .05). Nevertheless, we did find a statistically significant association between industry sponsorship and the exclusion of the no-vaccination strategy (*p* = .02), with sponsored studies excluding the no-vaccination strategy more often (*n* = 16/22; 73 percent) than independent research (*n* = 1/7; 14 percent). We found no association between sponsorship and the three assessment criteria (transparency of reporting, inclusion of relevant comparators, appropriateness of incremental comparisons; *p* = .58; *p* = .65; *p* = .24). We did find a highly statistically significant association between industry sponsorship and policy recommendations in the subset of studies that include both PCV10 and PCV13. All the economic evaluations sponsored by Pfizer concluded that PCV13 was the optimal strategy (*n* = 11/11; 100 percent), whereas nine out of ten studies (90 percent) sponsored by GSK found that PCV10 was cost-effective (*p* < .001).

## Discussion

Our analysis appraises the reliability of the policy recommendations of CEAs of infant pneumococcal immunization programs by examining the specific methodological issue of the comparisons made between strategies. Ostensibly, there is a large literature on pneumococcal vaccination and one might expect clear policy guidance to emerge. In practice, the studies are heterogeneous regarding the optimal policy choice. This appears, in part, due to the way strategies have been compared. We find very few studies have included a complete set of strategies and compared them appropriately.

Our findings show that only four (14 percent) of the twenty-nine CEAs adhered to economic evaluation guidelines regarding strategy choice, incremental comparisons performed, and outcomes reporting (29;34;42;48). This indicates there are disconcerting shortcomings regarding the reliability of CEA evidence regarding infant pneumococcal vaccination. The omission of relevant comparators is a prevalent problem in the reviewed studies. Of the twenty-nine CEAs, only five (17 percent) included all relevant intervention strategies (27;29;30;35;42). The omission of a given strategy might be justified if all prior analyses had shown it clearly to be dominated. Our study showed that there was no clear consensus within the existing literature that any strategy is always dominated, therefore the omission of any of the strategies examined is unjustified. Arguably, where the rationale for the exclusion of strategies is debatable, the presumption should be to include rather than omit strategies, thus avoiding concerns of bias stemming from incomplete comparator inclusion.

We also identified errors in the incremental comparisons of the identified CEAs. Eleven studies had relevant ICERs to report, of which four studies performed incorrect incremental comparisons. We also found simple failures to report outcomes completely, making it difficult to assess the appropriateness of comparisons in those cases, although this occurred only in a minority of studies. Additionally, some cases required additional calculations to determine the total costs of the intervention strategy. This finding is consistent with previous studies from Kauf (49) who also found poor reporting in CEAs for meningococcal vaccination in developed countries. This indicates that poor reporting persists as a basic problem. This is notable as transparency and reproducibility are fundamental pillars of scientific research. Moreover, we identified shortcomings within infant pneumococcal vaccination CEAs in a HIC context. Earlier studies have identified similar problems in other contexts. Both Saokaew et al. (8) and Zakiyah et al. (9) found limitations in comparator inclusion in published CEAs for childhood vaccinations in LMICs. Furthermore, our findings are also consistent with Kauf (49), which found that some CEAs failed to recognize the (in)efficiency of an intervention strategy.

Previous research on pneumococcal vaccination has also suggested that research sponsorship influences conclusions regarding the CE of immunization strategies (8). Our research echoes this, as a clear association between the most optimal vaccination strategy and sponsorship was identified. We found a statistically significant association between funding and no-vaccination strategy omission, as well as conclusions regarding the most optimal strategy. These results are also confirmed by a recent study from Zakiyah et al. (9), who reviewed pneumococcal evaluations in Asian contexts. They observed that many studies poorly described the role of the funder. As research sponsorship seems to have a significant impact on critical assumptions in CEA, and therefore CE conclusions, there is a need for more independent research.

Although methodological deviations within CEA is not a novel finding in general, the persistence of these inconsistencies in recent literature is concerning. Our analysis confirms that greater effort is still required to enhance methodological standards in CEA. Journal editors and reviewers need to familiarize themselves with the methods literature in order to adequately review submitted studies. Similarly, policy makers should be aware of the presence of inappropriate analyses and understand the correct method for health economic evaluations.

Better adherence to long-established methodological recommendations is required. To prevent overestimation of the efficiency of novel intervention strategies, all available strategies should be evaluated to recognize relevant comparators for evaluation. To this end, we recommend that all earlier developed strategies as well as the non-intervention strategy are considered for analysis. In addition, when an intervention is omitted due to the assumption it will be dominated, then this assumption should be made explicit to enhance the transparency and reliability of the research. Finally, in order to increase the transparency of reporting, the economic evaluation results should present the total estimated costs for each of the intervention options.

Our review has both limitations and strengths. There may be additional reasons for variation in costs and effects which have not been investigated in the present study including different disease prevalence, model types, cost assumptions, and other methodological considerations. Although we employed published methodological guidelines to inform our assessment of the reliability of CEAs, our categorization of the adequacy of comparisons naturally remains somewhat subjective. Additionally, the small number of observations formed a limitation for quantitative analysis. Nonetheless, our use of quantitative analyses has made the conclusions less subjective in nature.

## Conclusion

We identified frequent basic methodological shortcomings in CEAs of infant strategies against pneumococcal disease in HICs. The omission of potentially relevant vaccination strategies and a failure to make the correct incremental comparisons between strategies are common. We also found considerable heterogeneity in the study findings. This is apparently closely associated with study sponsorship. It is hoped our work will renew awareness of established methodological guidelines to improve continued academic research and prevent adoption of inefficient health policies. Our findings also highlight the need for more independently funded research to provide policy clarity.

## Supplementary material

For supplementary material accompanying this paper visit https://doi.org/10.1017/S0266462323000351. click here to view supplementary material
