# Supplementary material for: Appropriateness of strategy comparisons in cost-effectiveness analyses of infant pneumococcal vaccination: a systematic review
Source: Int J Technol Assess Health Care. 2023 Jul 12;39(1):e42. doi: 10.1017/S0266462323000351 (PMC11570002; doi:10.1017/S0266462323000351)
Supplement: Supplementary file 1 [file S0266462323000351sup.zip › S0266462323000351sup001.docx]

**Appendix I**

*Systematic Search Syntaxes*

| **Database** | **Search syntax** |
| --- | --- |
| Scopus | TITLE-ABS-KEY((pneumococcal OR pneumococcus) AND (vaccin* OR immunization OR immunisation) AND ("cost effect*" OR "cost utility"  OR "economic evaluation")) AND ALL(QALY* OR DALY* OR HALY* OR LYG* OR LYS* OR "quality adjusted life year" OR "disability adjusted life year" OR "health adjusted life year" OR "life year saved" OR "life year gained") AND PUBYEAR AFT 2009 AND (LIMIT-TO (DOCTYPE, "ar") OR LIMIT-TO (DOCTYPE, "re")) AND (LIMIT-TO (LANGUAGE, "English") |
| Embase | ((pneumococcal OR pneumococcus) AND (vaccin* OR immunization OR immunisation) AND ('cost effect*' OR 'cost utility' OR 'economic evaluation')):ab,ti AND (QALY* OR DALY* OR HALY* OR LYG* OR LYS* OR 'quality adjusted life year' OR 'disability adjusted life year' OR 'health adjusted life year' OR 'life year saved' OR 'life year gained') AND ([article]/lim OR [review]/lim) AND [english]/lim AND [2010-2022]/py |
| Web of Science | (TS=((pneumococcal OR pneumococcus) AND (vaccin* OR immunization OR immunisation) AND ("cost effect*" OR "cost utility" OR "economic evaluation")) AND ALL=(QALY* OR DALY* OR HALY* OR LYG* OR LYS* OR "quality adjusted life year" OR "disability adjusted life year" OR "health adjusted life year" OR "life year saved" OR "life year gained")) AND LANGUAGE: (English) AND DOCUMENT TYPES: (Article OR Review) Indexes=SCI-EXPANDED, SSCI, A&HCI, ESCI Timespan=2010-2022 |
| PubMed | ((pneumococcal [Title/Abstract] OR pneumococcus [Title/Abstract]) AND (vaccine [Title/Abstract] OR vaccination [Title/Abstract] OR immunization [Title/Abstract] OR immunisation [Title/Abstract]) AND ("cost effective" [Title/Abstract] OR "cost effectiveness" [Title/Abstract] OR "cost utility"[Title/Abstract] OR "economic evaluation" [Title/Abstract])) AND(QALY* [ALL FIELDS] OR DALY* [ALL FIELDS] OR HALY* [ALL FIELDS] OR LYG* [ALL FIELDS] OR LYS [ALL FIELDS] OR "quality adjusted life year" [ALL FIELDS] OR "disability adjusted life year" [ALL FIELDS] OR "health adjusted life year" [ALL FIELDS] OR "life year saved" [ALL FIELDS] OR "life year gained" [ALL FIELDS]) AND (Journal Article [ptyp] OR Review [ptyp]) AND ("2010/01/01" [PDat]:"2022/06/15" [PDat]) AND (English [lang]) |
