# Supplementary material for: Appropriateness of strategy comparisons in cost-effectiveness analyses of infant pneumococcal vaccination: a systematic review
Source: Int J Technol Assess Health Care. 2023 Jul 12;39(1):e42. doi: 10.1017/S0266462323000351 (PMC11570002; doi:10.1017/S0266462323000351)
Supplement: Supplementary file 1 [file S0266462323000351sup.zip › S0266462323000351sup002.docx]

**Appendix II**

*Pneumococcal Vaccine Development and Licensing Dates*

| **Licence date** | **Vaccine type** | **Serotypes included** |
| --- | --- | --- |
| 1977 | PPSV14: 14-valent pneumococcal polysaccharide vaccine for people 50 years and older or those 2 years and older with certain underlying health conditions | 1, 2, 3, 4, 5, 6A, 7F, 8, 9N, 12F, 18C, 19F, 23F, 25F |
| 1983 | PPSV23: 23-valent pneumococcal polysaccharide vaccine for adults and children older than two years of age ^a^ | 1, 2, 3, 4, 5, 6B, 7F, 8, 9N, 9V, 10A, 11A, 12F, 14,15B, 17F, 18C, 19F, 19A, 20, 22F, 23F, 33F |
| 2000 | PCV7: 7-valent pneumococcal  conjugate vaccine for infants under  5 years old | 4, 6B, 9V, 14, 18C, 19F, 23F |
| 2009 | PCV10: 10-valent pneumococcal conjugate vaccine for children under  5 years old | 1, 4, 5, 6B, 7F, 9V, 14, 18C, 19F, 23F |
| 2010 | PCV13: 13-valent pneumococcal conjugate vaccine for infants and young children between 6 months and 5 years | 1, 3, 4, 5, 6A, 6B, 7F, 9V, 14, 18C, 19A, 19F, 23F |
| 2011 | PCV13: 13-valent pneumococcal conjugate vaccine for adults 50 years  and older | 1, 3, 4, 5, 6A, 6B, 7F, 9V, 14, 18C, 19A, 19F, 23F |

**^a^** No protective immune response in children younger than 2 years of age
